# Correcting B_0_ Field Distortions in MRI Caused by Stainless Steel Orthodontic Appliances at 1.5 T Using Permanent Magnets – A Head Phantom Study

**DOI:** 10.1038/s41598-018-23890-6

**Published:** 2018-04-09

**Authors:** Zhiyue J. Wang, Yong Jong Park, Michael C. Morriss, Youngseob Seo, Trung Nguyen, Rami R. Hallac, Ana Nava, Rajiv Chopra, Yonatan Chatzinoff, Khyana Price, Nancy K. Rollins

**Affiliations:** 1 Department of Radiology, Children’s Health, Dallas, Texas USA; 2 Division of Orthodontics, Children’s Health, Dallas, Texas USA; 3 Analytical Imaging and Modeling Center, Children’s Health, Dallas, Texas USA; 4 Department of Radiology, University of Texas Southwestern Medical Center, Dallas, Texas USA; 5 Department of Plastic Surgery, University of Texas Southwestern Medical Center, Dallas, Texas USA; 6 Advanced Imaging Research Center, University of Texas Southwestern Medical Center, Dallas, Texas USA; 7 Center for Medical Metrology, Korea Research Institute of Standards and Science, Daejeon, Republic of Korea

## Abstract

Susceptibility artifacts caused by stainless steel orthodontic appliances (braces) pose significant challenges in clinical brain MRI examinations. We introduced field correction device (FCD) utilizing permanent magnets to cancel the induced B_0_ inhomogeneity and mitigate geometric distortions in MRI. We evaluated a prototype FCD using a 3D-printed head phantom in this proof of concept study. The phantom was compartmented into anterior frontal lobe, temporal lobe, fronto-parieto-occipital lobe, basal ganglia and thalami, brain stem, and cerebellum and had built-in orthogonal gridlines to facilitate the quantification of geometric distortions and volume obliterations. Stainless steel braces were mounted on dental models of three different sizes with total induced magnetic moment 0.15 to 0.17 A·m^2^. With braces B_0_ standard deviation (SD) ranged from 2.8 to 3.7 ppm in the temporal and anterior frontal lobes vs. 0.2 to 0.3 ppm without braces. The volume of brain regions in diffusion weighted imaging was obliterated by 32–38% with braces vs. 0% without braces in the cerebellum. With the FCD the SD of B_0_ ranged from 0.3 to 1.2 ppm, and obliterated volume ranged from 0 to 6% in the corresponding brain areas. These results showed that FCD can effectively decrease susceptibility artifacts from orthodontic appliances.

## Introduction

A large portion of the general population undergoes orthodontic treatment at some point in life, especially during adolescence. In this treatment, orthodontic appliances (commonly known as braces) are used to induce changes in teeth positions. The braces consist of orthodontic brackets that are affixed to teeth and arch wires engaging brackets to exert forces. The treatment improves oral function, occlusion and self-esteem and lasts from 6 months to over 2 years. Stainless steel orthodontic brackets and wires are most commonly used due to their effectiveness, durability and lower cost. Different types of stainless steel alloys are used in orthodontic appliances, depending on the mechanical property requirements.

Brain magnetic resonance imaging (MRI) is often acquired while orthodontic appliances are in place for a wide variety of medical conditions. Different MR sequences have differential sensitivity to susceptibility artifact. Diffusion imaging sequences used to screen for cytotoxic brain injury such as seen in stroke, MR angiography which provide non-invasive imaging of arteries, and MR spectroscopy which evaluates brain metabolites are particularly degraded by orthodontia-related artifacts. MRI relies on a highly uniform magnetic field to acquire images with diagnostic quality, but most stainless steel orthodontic appliances have large magnetic susceptibility that destroys field homogeneity and may render MR images non-diagnostic^1–3^. These artifacts pose significant challenges for diagnoses in clinical examinations. Emergent removal of orthodontic appliances is inconvenient, expensive, and not always possible and may delay MRI scanning. Repeatedly removing and replacing of orthodontia for serial surveillance MR imaging of chronic condition is impractical, leading to premature termination of orthodontic treatment.

The susceptibility artifacts manifest in MR imaging and spectroscopy in many different ways, including severe geometric distortions and obliteration of tissue volumes especially in techniques using EPI readout, excessive line broadening in MR spectroscopy, failure of chemical shift selective excitation or saturations, etc^4^. All of these problems can be addressed by restoring B_0_ field homogeneity.

Previously, intra-oral diamagnetic block or active shim coil were used to improve B_0_ homogeneity for brain fMRI in normal subjects^5,6^. Artifacts from orthodontia were too severe for these methods to be effective. We have developed a prototype field correction device (FCD) to restore the B_0_ field homogeneity with orthodontic appliances intact inside the mouth of patients. The FCD places small permanent magnets near the dental brackets with their magnetic moment in the opposite direction of that induced in the braces. If the magnetic moment of the device matches that in braces both in amplitude and spatial distribution, the field homogeneity theoretically could be restored to a level that enables MRI of diagnostic quality. In this report, we present quantitative results on the improvement of B_0_ homogeneity and geometric distortions using a FCD prototype on a head phantom.

## Results

**Figure 1:**
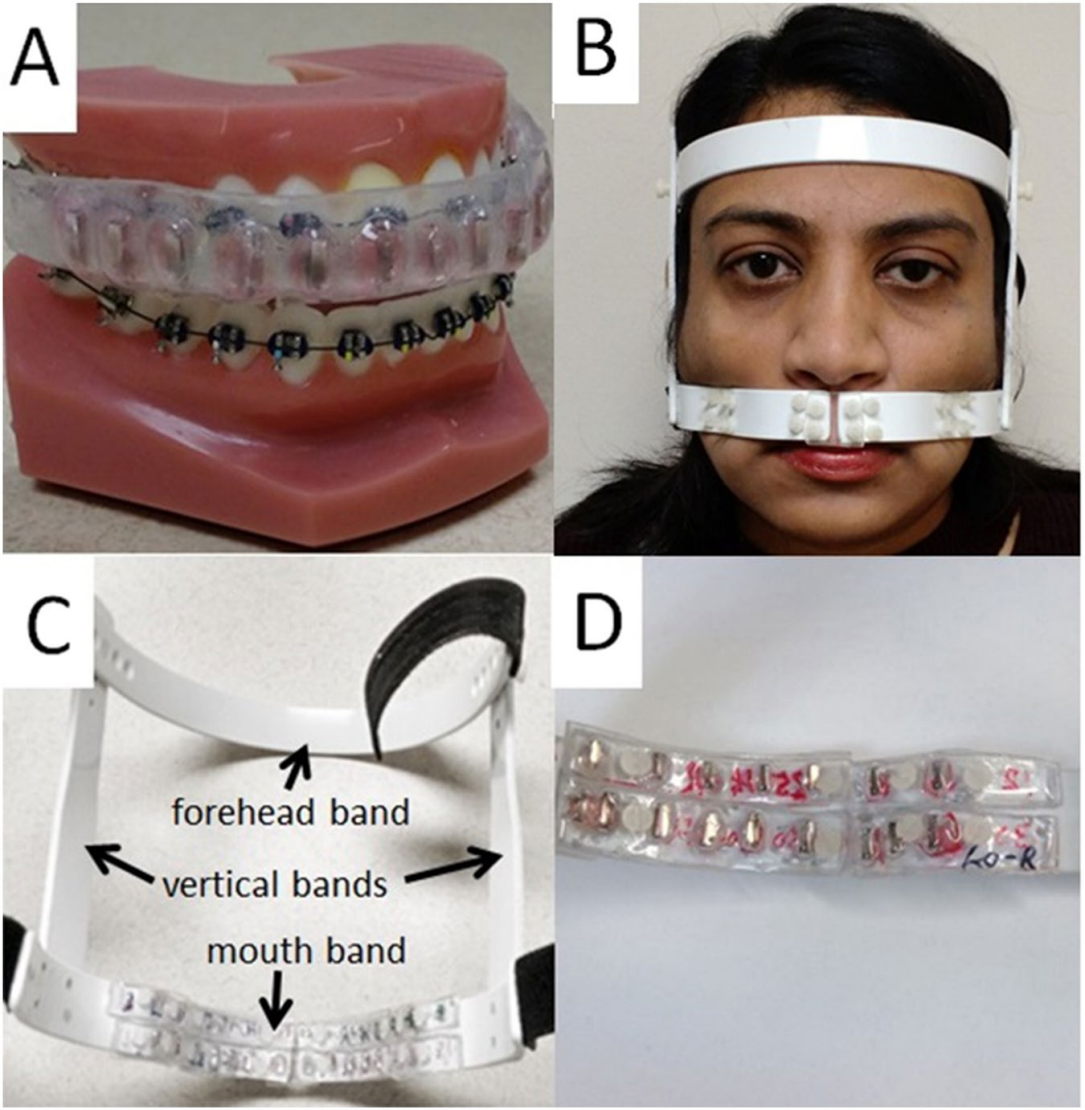
Field correction device. (**A**) Intra-oral device fitting on a dental model with braces. (**B**) External device fitting on the head. (**C**) Back view of the external device showing plastic strips mounted on the device. (**D**) Zoom-in back view on the right half of mouth-band showing 2 rows × 2 columns of plastic strips embedded with permanent magnets. The left half of mouth-band with other 2 rows × 2 columns of plastic strips is outside the view.

**Figure 2:**
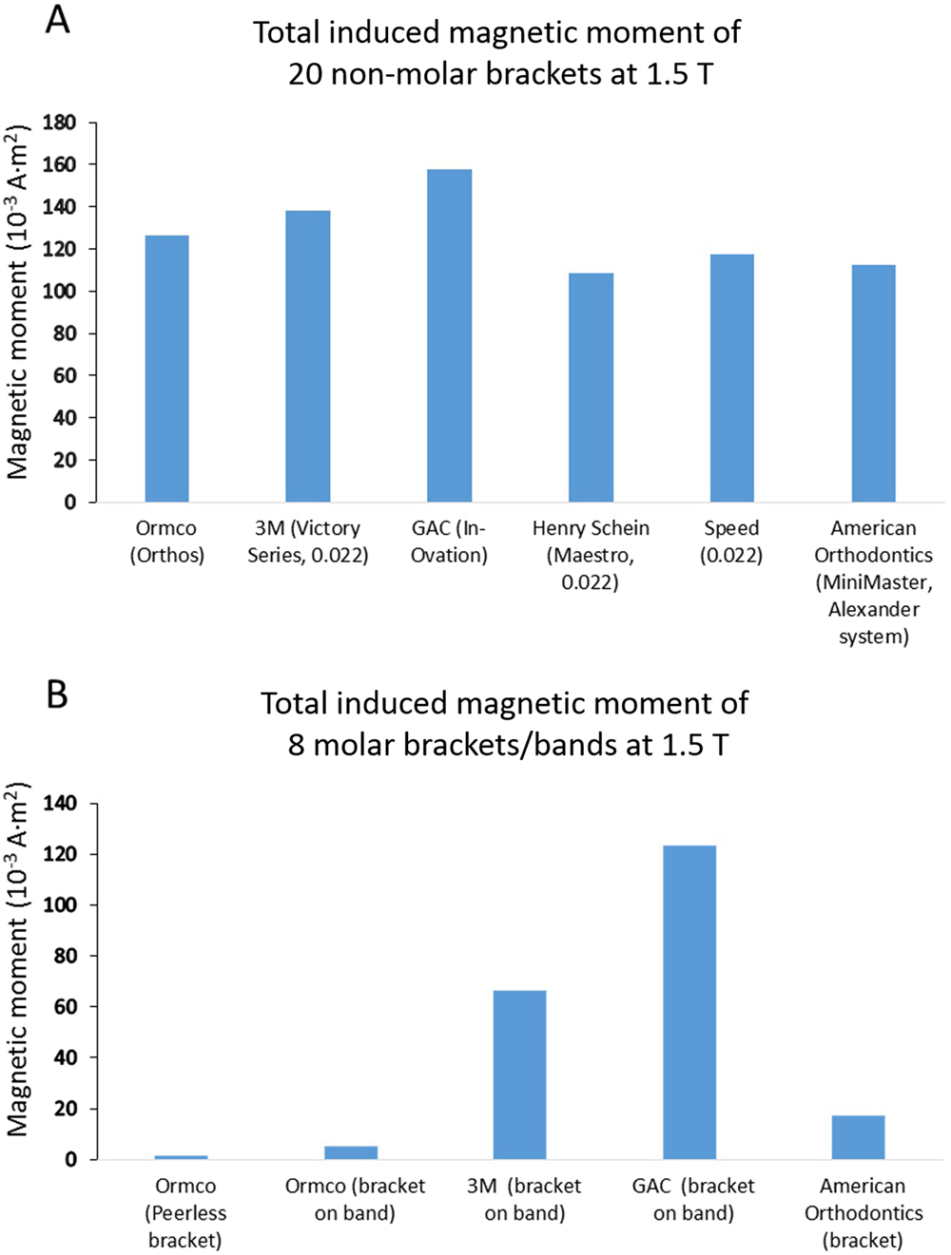
Induced magnetic moment in different orthodontic brackets inside a 1.5 T MRI scanner. The magnetic moment of individual stand-alone bracket or bracket on dental band was measured one at a time (ref.^13^) with a precision better than 10^−4^ A·m^2^. (**A**) Total magnetic moment of 20 non molar brackets (upper, lower, right and left central incisor, lateral incisor, canine, 1st premolar, 2^nd^ premolar) (ref.^13^). (**B**) Total magnetic moment of 8 molar (upper, lower, right, left 1st and 2^nd^ molar) brackets or brackets on bands. Data for the left 3 columns are from ref.^13^.

In this study, the effectiveness of FCD was evaluated using a 3D-printed head phantom. The prototype FCD consists of permanent magnets and plastic materials providing structural support. In order to minimize the susceptibility artifact, the magnetic moment of the FCD should approximate that of the braces both in amplitude and spatial distribution. The device has two main components, an intra-oral mouth-guard and an external face mask (Fig. 1). The intra-oral mouth-guard has permanent magnets embedded between two layers of acrylic thermoplastics. The external face-mask has strips of acrylic plastic embedded with magnets, which are mounted on the mouth-band of the face mask. Intra-oral mouth-guard provides close proximity between the correction magnets and the dental brackets but requires patient cooperation with biting to keep the device in place. The external device requires no effort from the patient since the device is attached to the head and immobilized using Velcro strips. Both intra-oral and external components have multiple exchangeable parts with different magnetic strengths in order to match orthodontic appliances of different vendors and models that have variable induced magnetic moment inside MRI scanners (Fig. 2). The intra-oral and external devices can be used together and match the total magnetic moment of common orthodontic appliances with an error of 10^−2^ A·m^2^ or less, as was done in this study.

**Figure 3:**
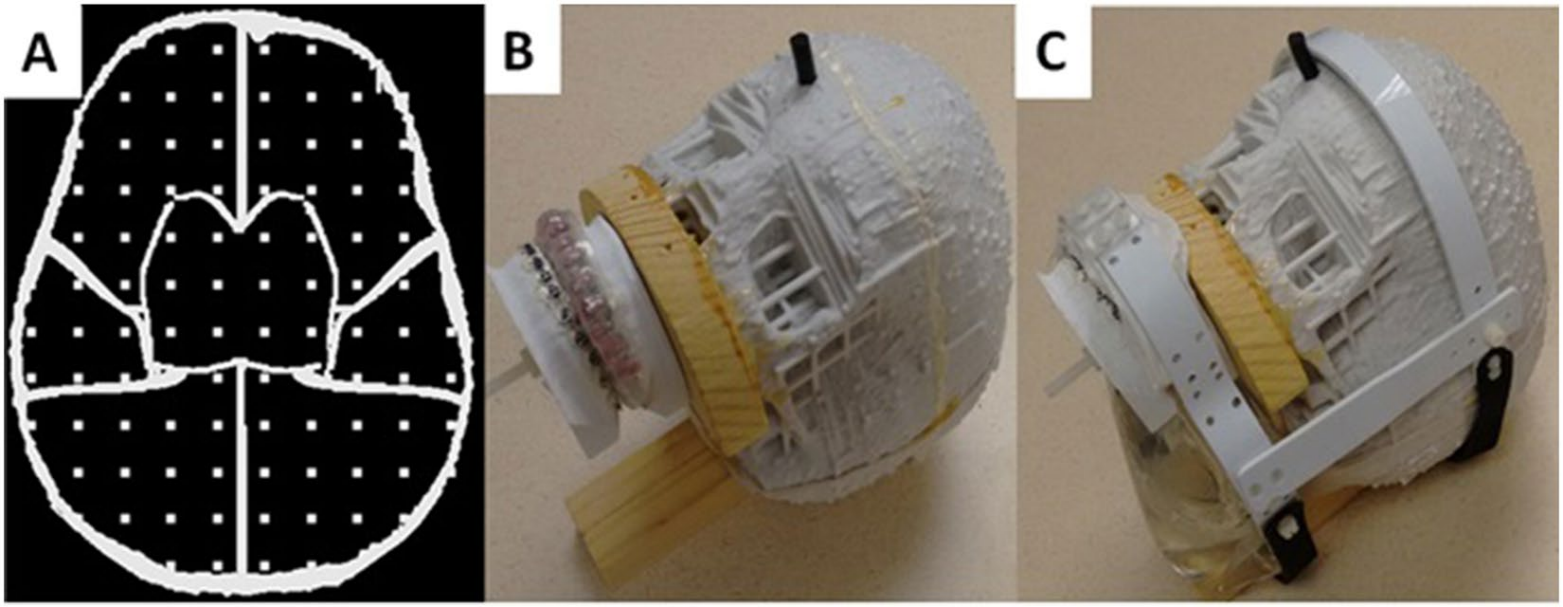
3D-printed head phantom. (**A**) Axial views of phantom design with compartments and a built-in grid. (**B**) Dental model and mouth-guard field correction device are mounted on the 3D-printed phantom. A rubber stopper is inserted in an opening on the forehead for filling the phantom with a solution. (**C**) External device is added, and a saline bag is added dorsal to the dental model to mimic the cervical soft tissues. The intra-oral device and external device are separated by 5 mm of paper mimicking the upper lip.

Head phantom and dental models were 3D printed (Fig. 3). The head phantom has the size of an average 15 years old. It included 9 compartments and had gridlines with 15 mm spacing along 3 orthogonal directions. Dental models with 28 teeth were made in 3 different sizes (small, medium and large). Stainless steel orthodontic brackets were bonded to each tooth and full length stainless steel arch-wires were engaged on the brackets. The measured magnetic moments of the 3 dental models and corresponding FCD configurations are listed in Table 1 where the intra-oral and external devices were used together. Experimental results using these configurations are reported.

**Table 1:** Estimated magnetic moment (10^−3^ A·m^2^) of orthodontic appliances and assembled FCD which uses the intra-oral and external components simultaneously.

	Magnetic moments	Small model	Medium model	Large model
Measured magnetic moments	On segments	[0, 74, 79, 0]^*^	[12, 65, 73, 11]^*^	[31, 46, 49, 43]^*^
	Total induced	153	161	169
Assembled device	Intra-oral	100	100	100
	External upper row	[0, 0, 0, 0]^*^	[16, 20, 20, 16]^*^	[0, 0, 0, 10]^*^
	External lower row	[0, 30, 30, 0]^*^	[0, 0, 0, 0]^*^	[30, 0, 0, 30]^*^
	Total matching	160	172	170

*Magnetic moment for left molars, left non-molars, right non-molars, right molars (see text).

MRI scans included 3D B_0_ mapping, axial spin-echo images as geometric reference and diffusion weighted imaging (DWI) using EPI readout. DWI is very important for evaluating stroke but sensitive to B_0_ inhomogeneity. These scans were acquired from the phantom under 4 conditions: (1) without braces and FCD; (2) with braces without FCD; (3) with braces and the FCD (intra-oral and external devices were used together); (4) same as (3) but tilting the chin up at 9 degrees.

**Figure 4:**
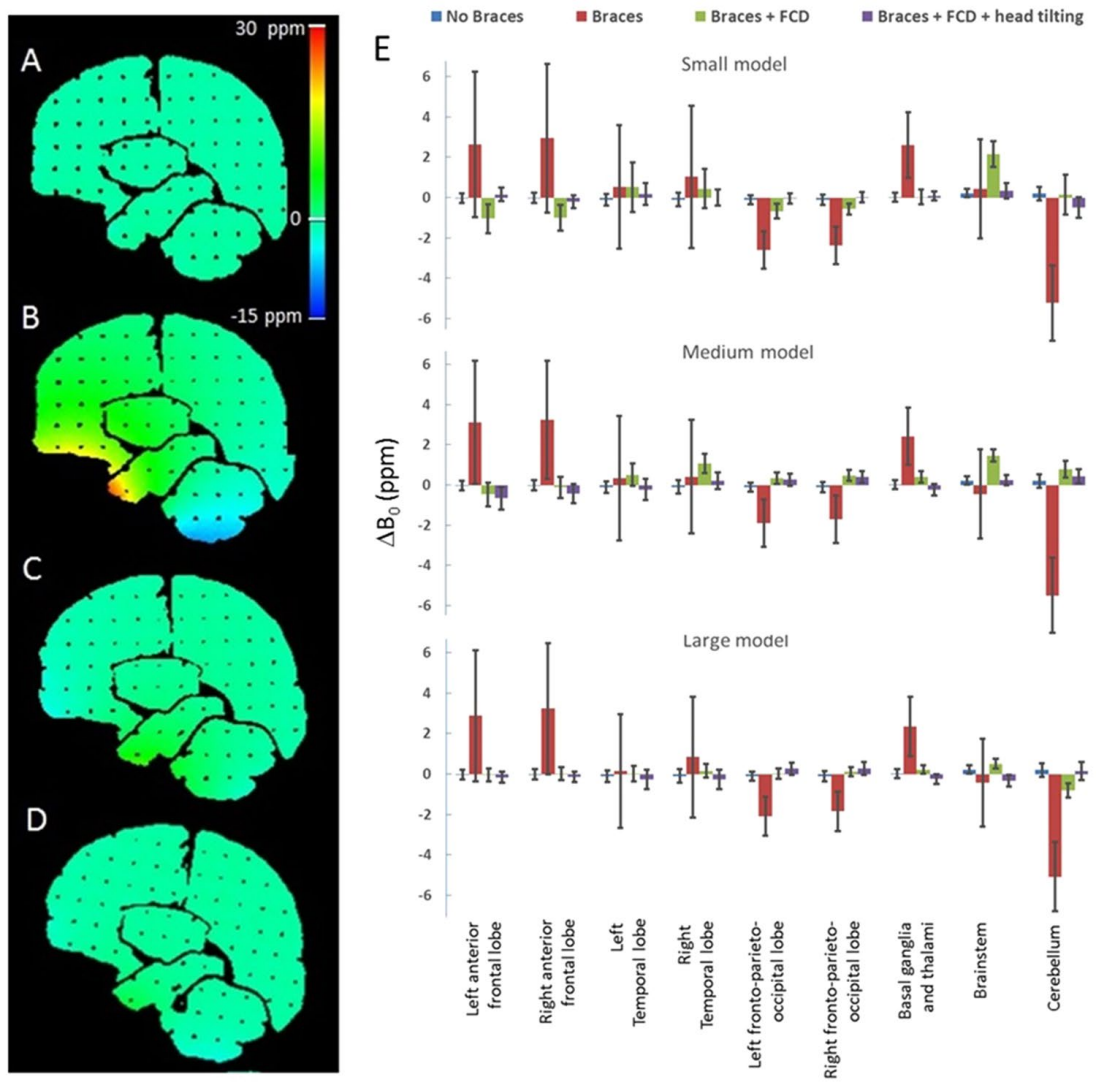
B_0_ field distribution inside the head phantom. (**A**) Baseline ΔB_0_ map when braces and FCD are absent. (**B**) The small dental model with orthodontic appliances is in place. (**C**) The small dental model with orthodontic appliances and FCD are both present. (**D**) Same as (**C**) but the phantom is tilted; (**E**) ΔB_0_ in brain compartments for three models of different sizes. The column height is the mean value and the error bar is ±SD. The average ΔB_0_ over the whole brain is set to zero.

We use ΔB_0_ to denote the inhomogeneous part of the B_0_ field. Results of the B_0_ homogeneity are shown in Figure 4. Fig. 4A–D show ΔB_0_ map in a sagittal slice. A baseline ΔB_0_ map is shown in Fig. 4A. Fig. 4B–D show ΔB_0_ map associated with the small dental model. Fig. 4E summarizes the results of all B_0_ measurements. Orthodontic appliances caused severe degradation in the anterior frontal lobe and temporal lobe without the FCD. The standard deviation of B_0_ ranging from 2.8 to 3.7 ppm in temporal and anterior frontal lobes vs. 0.2 to 0.3 ppm when the braces were absent. When the FCD was used, the standard deviation (SD) of B_0_ ranged from 0.3 to 1.2 ppm and improved by 84% on average in these regions.

**Figure 5:**
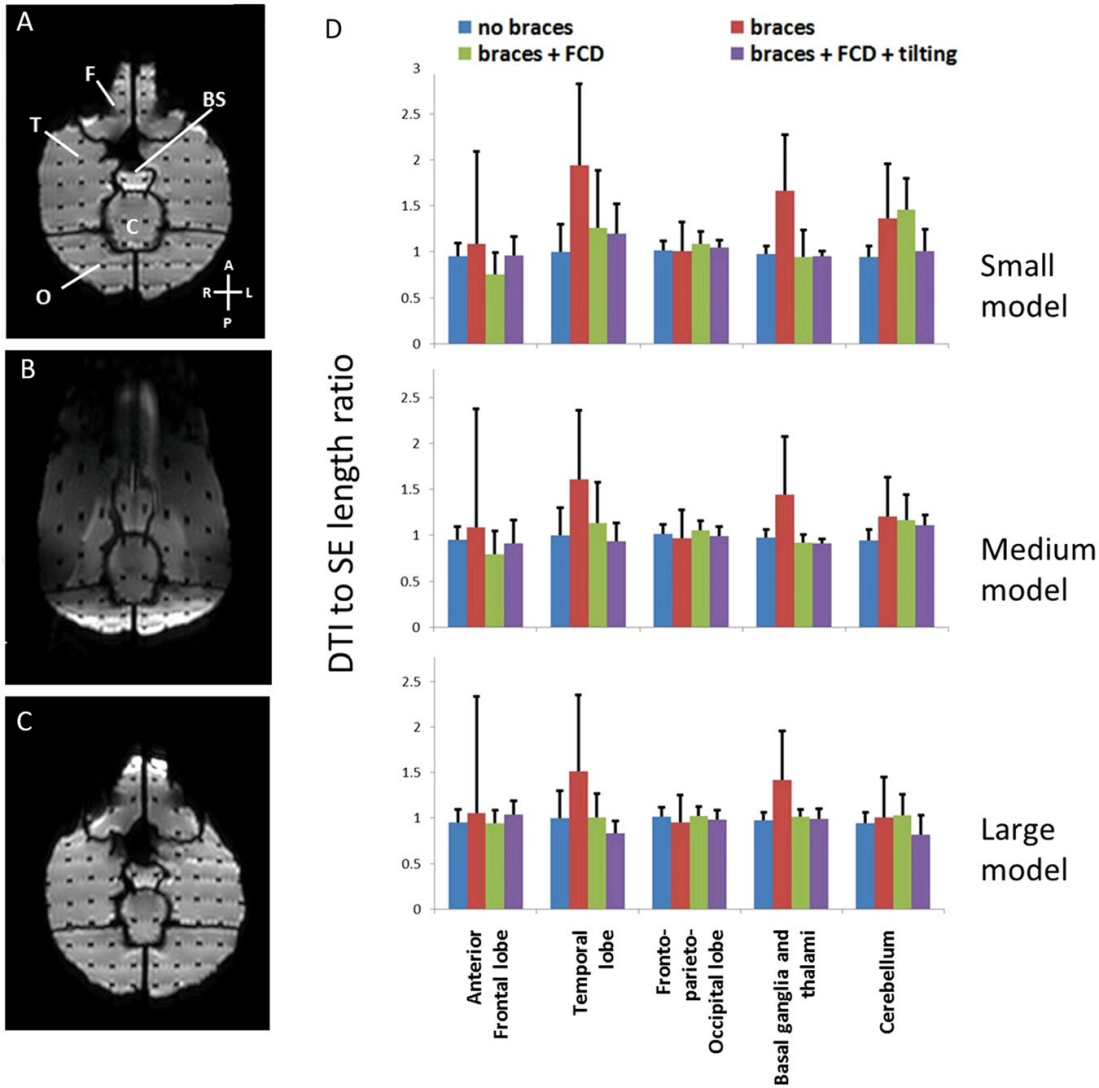
Quantitative assessment of geometric distortions in diffusion weighted imaging and quantitative assessment of distortions. An axial slice DWI of phantom (b = 0 image) is shown in (**A–C**). (**A**) Baseline image when dental braces were absent. F: frontal lobe; T: temporal lobe; BS: brainstem; C: cerebellum and O: occipital lobe. (**B**) Large dental braces model induced severe geometric distortions in the image and volume obliteration in frontal lobe. (**C**) Large dental braces model and correction device were both present. The geometric distortions were drastically decreased compared with (**B**) and the frontal lobe was visualized. (**D**) Length ratio (DWI: SE, mean (column height) + SD (error bar)) along AP direction in anterior frontal lobe, temporal lobe, fronto-parieto-occipital lobe, basal ganglia and thalami, and cerebellum are plotted for three model sizes under different conditions.

Figure 5A–C show an axial slice of DWI (b = 0) comparing a baseline scan (Fig. 5A), scan with the large braces model (Fig. 5B) and when the FCD was added (Fig. 5C). The frontal lobe areas were obliterated and geometric distortions throughout the slice were evident in Fig. 5B. The corrective effects of the FCD are shown in Fig. 5C. Fig. 5D shows the quantitative length ratio (grid line spacing measured on DWI divided by that on spin echo (SE) images, or DWI:SE as a short hand notation) for brain areas except for brainstem. The ratio is one if there is no distortion. In the figure the height of the column represents the average of length ratio, and the SD is shown as the error bar. The length ratio was not evaluated the brainstem because the range of coordinates was too small and linear regression did not produce meaningful results. In the temporal lobe, braces caused both an increased average length ratio (1.52 to 1.94) and large SD of the length ratio (0.75 to 0.84). In the anterior frontal lobe, the average length ratio was close to one, but there was a large SD (1.00 to 1.29) in the length ratio. When the FCD was used, there were drastic improvements in these metrics. The length ratio’s average (0.84 to 1.26) and SD (0.32–0.26) improved in temporal lobe. At interior frontal lobe, the SD of the length ratio improved as well (0.14 to 0.25). When examining these results, it should be noted that the length ratio analysis excluded areas of volume obliteration, where some of the most distorted areas were expected when no FCD was present.

Table 2 shows quantitative assessment of the obliterated volume in DWI. Volume obliteration was not observed when orthodontic appliances were absent. Orthodontic appliances obliterated signals in anterior frontal lobe, temporal lobe, brainstem and cerebellum but there was no loss in basal ganglia and thalami, as well as fronto-parieto-occipital lobes. Volume obliteration was most severe in the cerebellum (32% to 38%). When the FCD was used, the volume obliteration was greatly mitigated, with a worst case of 8% in the temporal lobes and the small model.

**Table 2:** Percent volume obliteration in DWI in different regions of the head phantom.

	anterior frontal lobes	temporal lobes	fronto-parieto-occipital lobes	Basal ganglia and thalami	Brain-stem	Cere-bellum
Baseline (no braces)	0	0	0	0	0	0
Small model braces	21	19	0	0	14	33
Medium model braces	17	19	0	0	17	38
Large model braces	18	14	0	0	17	32
Small model braces/correction	3	8	0	0	0	0
Medium model braces/correction	3	1	0	0	0	6
Large model braces/correction	0	0	0	0	0	0
Small model braces/correction/tilting	2	2	0	0	0	0
Medium model braces/correction/tilting	2	3	0	0	0	2
Large model braces/correction/tilting	0	0	0	0	0	0

## Discussion

Susceptibility artifacts induced by stainless steel orthodontic appliances may interfere with clinical diagnoses during brain MRI scans. Both software and hardware correction approaches may be used. Pulse sequences may be made less prone to susceptibility effects and to decrease geometric distortions^7–9^. For example, MRI vendors offers scanning features such as WARP or OMAR which combine high bandwidth signal acquisition with turbo spin echo utilizing VAT^7^ and/or SEMEC^9^. This approach requires modification of each individual pulse sequence, but not all sequence can be readily modified to overcome the artifact. In addition, trade-offs are often made including longer acquisition times, decreased SNR and increased SAR levels. The hardware approach presented here addresses the problem at its root. However, matching the device to each individual patient is crucial. A device kit would consist of several exchangeable intra-oral mouth-guards with different magnetic strengths, and exchangeable parts with different magnetic strength that can be mounted on positions on the external face mask. For a particular patient, the device will be assembled by choosing one device piece in each position based on an analysis of the B_0_ map from a calibration MRI scan. Usually, this can be done in about 15 minutes. In addition, safety concerns and risk factors for using the device need to be addressed.

The field correction device demonstrated here works for 1.5 T MRI scanners. The NdFeB permanent magnets used in the devices have an intrinsic coercivity of about 2140 kA/m (corresponds to 2.7 T) at the body temperature and resist irreversible demagnetization inside 1.5 T MRI scanners. The permanent magnets are very stable. The magnets have 10 years of shelf life and may be remagnetized periodically by strong external magnetic field. During the MRI session, the magnetization was found to be stable^10^. Permanent magnets with higher intrinsic coercivity than the ones used here are now available and could be used for FCD at 3 T MRI scanners. At 3 T, the induced magnetic moment in orthodontic appliances will be larger than that at 1.5 T. Consequently, the FCD will need to have a larger total magnetic moment in order to counter the induced magnetic moment in braces. The FCD experience the largest net force at the entrance of the MRI magnet where the B_0_ gradient is large. At the center of the magnet the B_0_ gradient is very small, so the net force is approximately zero. Furthermore, at the center of the magnet, there is a torque on the device if the magnetic moment of the device is not perfectly anti-parallel to the B_0_ field. The torque experienced by the device will be proportional to the cross product of total magnetic moment and the external magnetic field, and the force on the device will be proportional to the dot product of total magnetic moment and field gradient. Both magnetic torque and force will be larger at 3 T. Therefore, working at 1.5 T is more favorable from a safety perspective.

Previous presentations at scientific conferences have shown research studies using the device in patients or research participants undergoing orthodontic treatment^11,12^, where the use of FCD effectively restored B_0_ homogeneity, removed large geometric distortions on diffusion weighted images and allowed uncompromised visualization of blood vessels on MR angiography. In addition, the device was well tolerated by subjects during the MRI scans. The phantom work in the current study enabled quantitative assessment of B_0_ homogeneity and geometric distortion systematically. In evaluating FCD, assessing B_0_ field homogeneity is important because the artifacts on an MR image or spectrum from a pulse sequence can be predicted, at least in principle, if the B_0_ field distribution is known. This phantom study demonstrates the effectiveness of a FCD in decreasing the severity of artifacts. The intra-oral mouth-guard and external face mask have one size, yet the artifacts from dental models of different sizes can be corrected.

This is a proof-of-concept study, so the testing was limited in scale. In patient examinations, there are many variables that may affect the performance of the device. The effectiveness of the device also needs to be studied for more MRI techniques including MR angiography, susceptibility weighted imaging, diffusion tensor imaging and functional MRI. The current phantom is not optimal for these studies. Ultimately the effectiveness of the device will need to be tested in a patient population wearing all types of orthodontic appliances on a variety of MRI techniques and in multicenter clinical studies.

The prototype intra-oral device built for this study was intended to be used in combination with the external device. The intra-oral kit has a few exchangeable pieces with different magnetic moment, but the increment of magnetic moment is relatively coarse. In addition, the intra-oral device presented here was not designed for correcting the B_0_ inhomogeneity caused by molar orthodontic brackets. Therefore, generally the intra-oral component is not suitable to be used by itself. The external device may be used by itself. In this case the average distance between the orthodontic brackets and correction magnets will slightly increase. However, we do not expect drastic degradation of the effectiveness of field correction.

The prototype device design is currently being improved so it will be more comfortable, more user-friendly, and safer to use.

## Conclusions

The study provides quantitative evidence that a field correction device can effectively improve B_0_ homogeneity and geometric distortions in MRI caused by stainless steel orthodontic appliances.

## Materials and Methods

This study did not involve human or animals. No biological materials were used. The study was performed within the privilege of the authors granted by Children’s Medical Center Dallas and/or University of Texas Southwestern Medical Center. The study protocol was not subject to institutional review.

### Induced magnetic moment in orthodontic appliances

The numerous vendors of orthodontic appliances offer ever changing diverse products. Stand-alone orthodontic brackets are used for incisor, canine and premolar teeth. For molars, brackets may be stand alone as on non-molar teeth or attached to an orthodontic band which in turn is mounted on teeth. Arch wires also possess induced magnetic moment although dominant contributions come from orthodontic brackets and bands. The orthodontic brackets and bands from different vendors differ in weight, shape, material composition, and induced magnetic moment inside the MRI scanner^13^. Fig. 2 shows the magnetic moment of non-molar and molar brackets made by several vendors. As can be seen from Fig. 2A, there is a factor of 1.6 variability of the total magnetic moment for commonly used brackets on non-molar teeth. The variability of the magnetic moment on the molar brackets or bands is larger, and the magnetic moment can be weak or strong compared with non-molar brackets.

### Permanent magnet employed in field correction device

We used commercial NdFeB^14^ permanent magnets (grade N38EH, Dexter Magnetics) which have a strong intrinsic coercivity of 2140 kA/m (corresponding to 2.7 T B field in vacuum) at body temperature and resist irreversible demagnetization at 1.5 T. The maximum energy product is sufficiently high, so small magnets can cancel out the induced magnetic moments in braces.

The magnets were coated with a thin layer of nickel and were custom made to specified sizes (Table 3). For each size, the magnetic moment of several samples was measured inside 1.5 T MRI scanner using method described previously^13^. The magnetic moment was anti-parallel to the B_0_ field during the measurement. The mean and standard deviation (SD) measured in 4 magnets of each size is also included in Table 3.

**Table 3:** Magnetic moments (mean ± SD, n = 4) of N38EH magnets with magnetization opposing 1.5 T B_0_ field (H_0_ = −1197 kA/m).

Magnet dimensions (mm^3^)	Magnetic moment (10^−3^ A·m^2^)
0.65 × 0.65 × 2.5	0.61 ± 0.11
1.0 × 1.0 × 3.9	2.79 ± 0.19
1.1 × 1.1 × 4.2	3.93 ± 0.06
1.2 × 1.2 × 4.6	5.00 ± 0.19
1.3 × 1.3 × 5.0	6.17 ± 0.11
1.4 × 1.4 × 5.4	7.97 ± 0.30
1.5 × 1.5 × 5.8	10.67 ± 0.17

### Design of field correction device

In order to minimize the susceptibility artifact, the magnetic moment of the FCD should approximate that of the braces both in amplitude and spatial distribution. The intra-oral device was constructed with FDA approved commercial dental acrylic thermoplastics (Great Lake Orthodontics) using a vacuum former. A layer of plastic (1 mm thickness) was molded on a dental upper arch template. Permanent magnets (grade N38EH, Dexter Magnetics) were glued (Triad Gel, Densply) to the plastic in locations corresponding to incisor, canine and premolar teeth. Another layer of plastic (2 mm thickness) was molded over the first layer. The device was magnetized using a 9.4 T MRI scanner. Acrylic welding solvent (Weld-On 4, IPS Corporation) was applied at the exposed interface of the two plastic layers at the edge of the device, resulting in strong bonding between the two layers.

The frame of the external device is constructed using PVC plastic. The material becomes soft and deformable at an elevated temperature (about 80 °C) and is rigid at room and body temperatures. The plastic strips embedding the permanent magnets were also made with a vacuum former. The strips are rigid, and mounted on the external device in 2 rows and 4 columns using nylon screws and nuts. Two central columns correct the induced magnetic moments of brackets on incisor, canine and premolar teeth, and two lateral columns correct that of brackets on molars. These magnetic strips can be mounted on the mouth-band of the face mask in a few minutes. The external device offers the flexibility for correcting artifacts from unilateral implants as well.

The intra-oral and external devices were constructed using permanent magnets listed in Table 3. Using magnets with different magnetic moments, device pieces with different nominal magnetic moments were constructed. The magnetic moment was distributed approximately evenly across the intended length. A device kit has multiple pieces for intra-oral mouth-guard with total magnetic moment up to 150 × 10^−3^ A·m^2^, and there are multiple strips for the external facemask with different magnetic moment accommodating a total magnetic moment up to 366 × 10^−3^ A·m^2^.

### Dental model and head phantom

Maxillary and mandibular dental models with 14 teeth were 3D printed (ZPrinter 650 and zp150 composite powder from Z-Corporation) from digital files (OrthoCad). The models were made in 3 sizes (the original size presenting the average size of the general population, and 8.5% smaller and 8.5% larger in linear dimension). A set of 28 orthodontic brackets (American Orthodontics, slot size 0.022″) were bonded onto the dental models. Full length stainless steel arch-wires (Class One, 0.016″ diameter) were engaged on the brackets. The dental model was mounted on the head phantom using a wood spacer and a nylon screw.

A human head phantom equivalent in size to an average 15 years old^15^ was 3D printed (ZPrinter 650 and zp150 composite powder from Z-Corporation) (Fig. 3). The head circumference of the phantom was 54.6 cm. The phantom was divided into 9 compartments: left and right anterior frontal lobes (2 compartments), left and right temporal lobes (2 compartments), left and right fronto-parieto-occipital lobes (2 compartments), basal ganglia and thalami (1 compartment), brainstem (1 compartment) and cerebellum (1 compartment). It had built-in orthogonal gridlines along 3 directions with the nearest line distance of 15 mm. This phantom was filled with saline (distilled water and 0.9% NaCl by weight) and doped with an MRI contrast agent (1 mL ProHance per liter saline). When a dental model was mounted on the head phantom, the vertical distance between the tip of the upper incisor and nasion was 70 mm. A bag of saline (400 mL) was positioned inferior to the head phantom and posterior to the dental model to mimic the cervical soft tissues.

### MRI scanning

The MRI scans were acquired on an Achieva 1.5 T MRI scanner (Software release 2.6.1, Philips Healthcare) using an eight channel SENSE head coil. The scans included (i) 3D B_0_ mapping (3D sagittal T1-FFE, TR = 10 ms, acquisition voxel 2 × 2 × 2 mm^3^, acquisition array size 70(RL) × 112(AP) × 112(HF), 2 acquisitions with different TEs at 3.00 ms and 3.07 or 3.20 ms), (ii) Multi-slice spin-echo (SE) imaging as a geometrical reference (FOV = 250 mm, TR/TE = 479/12 ms, slice thickness = 4.0 mm, gap = 0.4 mm, number of slices = 35, water-fat shift = 1.5 mm in AP direction), and (iii) diffusion weighted imaging (DWI) using EPI readout (FOV = 250 mm, TR/TE = 4682/74 ms, slice thickness = 4.0 mm, gap = 0.4 mm, number of slices = 35, water-fat shift of 33 mm in the AP direction, b = 0, 1000 mm^2^/s). Diffusion-weighted imaging is particularly important for examination of stroke^16,17^.

### Matching field correction device to orthodontic appliances

All analyses were done using internally developed software written in IDL 8.4. The ΔB_0_ map was calculated from the difference of T1-FFE phase images. The ΔB_0_ map with braces was analyzed to determine the strength and distribution of the induced magnetic moment of orthodontic appliances. The 3D ΔB_0_ map was modeled by the z-component magnetic field from 14 diploes symmetrically positioned on the maxillary arch. By doing so we treated the brackets on the corresponding upper and lower teeth as one magnetic dipole. The 14 dipoles were further divided into 4 segments: lateral segments for left and right molars and central segments for left and right non-molars. The lateral segments corresponded to the two outer columns on the external device, and the two central segments corresponded to the intra-oral device and the two inner columns on the external device. Within each segment the magnetic moment is represented by one fitting parameter assuming equal magnetic moment of the orthodontic brackets. The relative positions of the fitting dipoles are fixed and translation of all dipoles together as a whole is allowed in model fitting. Thus, a least squares procedure was used to fit the B_0_ field measured with a braces model using 7 parameters (3 parameters for displacement and 4 parameters for magnetic moment of each segment). The B_0_ analysis takes less than 30 seconds to complete. The resulting magnetic moments on the 4 segments were used to guide the assembling of the device to match the braces. The magnetic moment of the two outer segments should be matched by the two outer columns of the external device. The magnetic moment of the two inner segments should be matched by the two inner segments on the external device and the intra-oral device. In Table 1, the magnetic moment on each segment is matched with an error from 0 to 6 × 10^−3^ A·m^2^, and the total magnetic moment was matched with an error from 1 × 10^−3^ to 11 × 10^−3^ A·m^2^

### Image quality analysis

All analyses were done using internally developed software written in IDL 8.4 unless otherwise stated. MR images were processed to obtain the average and SD of B_0_ and geometric distortion on DWI for brain compartments. Geometric distortions were quantified by linear regression of positions of intercepting points in AP direction in the 3D grids on the SE (as independent variable) and DWI (as dependent variable) in brain regions except for brainstem where the range of position is too small for reliable results. The linear regression was done using Microsoft Excel. The slope of the regression line was the average length ratio (DWI:SE), and the SD of the length ratio was obtained from the residual error divided by the gridline spacing (15 mm). Finally, the percent of obliterated volume on DWI was also estimated in these brain regions by counting and comparing the number of visualized grid intersections in these regions on DWI and SE images. In the analysis of geometric distortions and volume obliteration the corresponding left and right hemispheric lobes were combined because the results from two sides were similar although B_0_ homogeneity for each side were reported separately.

### Data Availability

MR images from this study are available to interested readers upon reasonable request.
